# Efficient sequential and parallel algorithms for finding edit distance based motifs

**DOI:** 10.1186/s12864-016-2789-9

**Published:** 2016-08-18

**Authors:** Soumitra Pal, Peng Xiao, Sanguthevar Rajasekaran

**Affiliations:** 1 Department of Computer Science and Engineering, University of Connecticut, 371 Fairfield Road,, Storrs, 06269 CT USA; 2 Department of Computer Science and Engineering, University of Connecticut, 371 Fairfield Road,, Storrs, 06269 CT USA

**Keywords:** Motif, Edit distance, Trie, Radix sort

## Abstract

**Background:**

Motif search is an important step in extracting meaningful patterns from biological data. The general problem of motif search is intractable and there is a pressing need to develop efficient, exact and approximation algorithms to solve this problem. In this paper, we present several novel, exact, sequential and parallel algorithms for solving the (*l,d*) *Edit-distance-based Motif Search (EMS)* problem: given two integers *l,d* and *n* biological strings, find all strings of length *l* that appear in each input string with atmost *d* errors of types substitution, insertion and deletion.

**Methods:**

One popular technique to solve the problem is to explore for each input string the set of all possible *l*-mers that belong to the *d*-neighborhood of any substring of the input string and output those which are common for all input strings. We introduce a novel and provably efficient neighborhood exploration technique. We show that it is enough to consider the candidates in neighborhood which are at a distance exactly *d*. We compactly represent these candidate motifs using wildcard characters and efficiently explore them with very few repetitions. Our sequential algorithm uses a trie based data structure to efficiently store and sort the candidate motifs. Our parallel algorithm in a multi-core shared memory setting uses arrays for storing and a novel modification of radix-sort for sorting the candidate motifs.

**Results:**

The algorithms for EMS are customarily evaluated on several challenging instances such as (8,1), (12,2), (16,3), (20,4), and so on. The best previously known algorithm, EMS1, is sequential and in estimated 3 days solves up to instance (16,3). Our sequential algorithms are more than 20 times faster on (16,3). On other hard instances such as (9,2), (11,3), (13,4), our algorithms are much faster. Our parallel algorithm has more than 600 % scaling performance while using 16 threads.

**Conclusions:**

Our algorithms have pushed up the state-of-the-art of EMS solvers and we believe that the techniques introduced in this paper are also applicable to other motif search problems such as Planted Motif Search (PMS) and Simple Motif Search (SMS).

**Electronic supplementary material:**

The online version of this article (doi:10.1186/s12864-016-2789-9) contains supplementary material, which is available to authorized users.

## Background

Motif search has applications in solving such crucial problems as identification of alternative splicing sites, determination of open reading frames, identification of promoter elements of genes, identification of transcription factors and their binding sites, etc. (see e.g., Nicolae and Rajasekaran [1]). There are many formulations of the motif search problem. A widely studied formulation is known as (*l,d*)-motif search or Planted Motif Search (PMS) [2]. Given two integers *l,d* and *n* biological strings the problem is to find all strings of length *l* that appear in each of the *n* input strings with atmost *d* mismatches. There is a significant amount of work in the literature on PMS (see e.g., [1, 3–5], and so on).

PMS considers only point mutations as events of divergence in biological sequences. However, insertions and deletions also play important roles in divergence [2, 6]. Therefore, researchers have also considered a formulation in which the Levenshtein distance (or edit distance), instead of mismatches, is used for measuring the degree of divergence [7, 8]. Given *n* strings *S*^(1)^,*S*^(2)^,…,*S*^(*n*)^, each of length *m* from a fixed alphabet *Σ*, and integers *l,d*, the *Edit-distance-based Motif Search (EMS)* problem is to find all patterns *M* of length *l* that occur in atleast one position in each *S*^(*i*)^ with an edit distance of atmost *d*. More formally, *M* is a motif if and only if ∀*i*, there exist *k*∈ [ *l*−*d,l*+*d*],*j*∈ [ 1,*m*−*k*+1] such that for the substring $S^{(i)}_{j,k}$ of length *k* at position *j* of *S*^(*i*)^, $ED\left (S^{(i)}_{j,k},M\right) \le d$. Here *ED*(*X,Y*) stands for the edit distance between two strings *X* and *Y*.

EMS is also NP-hard since PMS is a special case of EMS and PMS is known to be NP-hard [9]. As a result, any exact algorithm for EMS that finds all the motifs for a given input can be expected to have an exponential (in some of the parameters) worst case runtime. One of the earliest EMS algorithms is due to Rocke and Tompa [7] and is based on Gibbs Sampling which requires repeated searching of the motifs in a constantly evolving collection of aligned strings, and each search pass requires *O*(*nl*) time. This is an approximate algorithm. Sagot [8] gave a suffix tree based exact algorithm that takes *O*(*n*^2^*ml*^*d*^|*Σ*|^*d*^) time and *O*(*n*^2^*m*/*w*) space where *w* is the word length of the computer. Adebiyi and Kaufmann [10] proposed an exact algorithm with an expected runtime of *O*(*nm*+*d*(*nm*)^(1+*pow*(*ε*))^ log*nm*) where *ε*=*d*/*l* and *pow*(*ε*) is an increasing concave function. The value of *pow*(*ε*) is roughly 0.9 for protein and DNA sequences. Wang and Miao [11] gave an expectation minimization based heuristic genetic algorithm.

Rajasekaran et al. [12] proposed a simpler Deterministic Motif Search (DMS) that has the same worst case time complexity as the algorithm by Sagot [8]. The algorithm generates and stores the neighborhood of every substring of length in the range [*l*−*d,l*+*d*] of every input string and using a radix sort based method, outputs the neighbors that are common to atleast one substring of each input string. This algorithm was implemented by Pathak et al. [13].

Following a useful practice for PMS algorithms, Pathak et al. [13] evaluated their algorithm on certain instances that are considered challenging for PMS: (9,2), (11,3), (13,4) and so on [1], and are generated as follows: *n*=20 random DNA/protein strings of length *m*=600, and a short random string *M* of length *l* are generated according to the independent identically distributed (i.i.d) model. A separate random *d*-hamming distance neighbor of *M* is “planted” in each of the *n* input strings. Such an (*l,d*) instance is defined to be a *challenging instance* if *l* is the largest integer for which the expected number of spurious motifs, *i.e.*, the motifs that would occur in the input by random chance, is atleast 1.

The expected number of spurious motifs in EMS are different from those in PMS. Table 1 shows the expected number of spurious motifs for *l*∈ [ 5,21] and *d* upto max{*l*−2,13}, *n*=20, *m*=600 and *Σ*={*A,C,G,T*} [see Additional file 1]. The challenging instances for EMS turn out to be (8,1), (12,2), (16,3), (20,4) and so on. To compare with [13], we consider both types of instances, specifically, (8,1), (9,2), (11,3), (12,2), (13,4) and (16,3).

**Table 1:** Expected number of spurious motifs in random instances for *n*=20,*m*=600. Here, *∞* represents value ≥1.0*e*+7

*l*	*d*=0	1	2	3	4	5	6	7	8	9	10	11	12	13
5	0.0	1024.0	1024.0	*∞*										
6	0.0	4096.0	4096.0	*∞*	*∞*									
7	0.0	14141.8	16384.0	*∞*	*∞*	*∞*								
8	0.0	**225.8**	65536.0	65536.0	*∞*	*∞*	*∞*							
9	0.0	0.0	262144.0	262144.0	*∞*	*∞*	*∞*	*∞*						
10	0.0	0.0	1047003.6	1048576.0	*∞*	*∞*	*∞*	*∞*	*∞*					
11	0.0	0.0	1332519.5	4194304.0	*∞*	*∞*	*∞*	*∞*	*∞*	*∞*				
12	0.0	0.0	**294.7**	1.678e+07	1.678e+07	*∞*	*∞*	*∞*	*∞*	*∞*	*∞*			
13	0.0	0.0	0.0	6.711e+07	6.711e+07	*∞*	*∞*	*∞*	*∞*	*∞*	*∞*	*∞*		
14	0.0	0.0	0.0	2.517e+08	2.684e+08	*∞*	*∞*	*∞*	*∞*	*∞*	*∞*	*∞*	*∞*	
15	0.0	0.0	0.0	2.749e+07	1.074e+09	*∞*	*∞*	*∞*	*∞*	*∞*	*∞*	*∞*	*∞*	*∞*
16	0.0	0.0	0.0	**139.1**	4.295e+09	4.295e+09	*∞*	*∞*	*∞*	*∞*	*∞*	*∞*	*∞*	*∞*
17	0.0	0.0	0.0	0.0	1.718e+10	1.718e+10	*∞*	*∞*	*∞*	*∞*	*∞*	*∞*	*∞*	*∞*
18	0.0	0.0	0.0	0.0	3.965e+10	6.872e+10	*∞*	*∞*	*∞*	*∞*	*∞*	*∞*	*∞*	*∞*
19	0.0	0.0	0.0	0.0	1.226e+08	2.749e+11	2.749e+11	*∞*	*∞*	*∞*	*∞*	*∞*	*∞*	*∞*
20	0.0	0.0	0.0	0.0	**35.8**	1.100e+12	1.100e+12	*∞*	*∞*	*∞*	*∞*	*∞*	*∞*	*∞*
21	0.0	0.0	0.0	0.0	0.0	4.333e+12	4.398e+12	*∞*	*∞*	*∞*	*∞*	*∞*	*∞*	*∞*

The instances in bold represents challenging instances

The sequential algorithm by Pathak et al. [13] solves the moderately hard instance (11,3) in a few hours and does not solve the next difficult instance (13,4) even after 3 days. A key time-consuming part of the algorithm is in the generation of the edit distance neighborhood of all substrings as there are many common neighbors.

### Contributions

In this paper we present several improved algorithms for EMS that solve instance (11,3) in less than a couple of minutes and instance (13,4) in less than a couple of hours. On (16,3) our algorithm is more than 20 times faster than EMS1. Our algorithm uses an efficient technique (introduced in this paper) to generate the edit distance neighborhood of length *l* with distance atmost *d* of all substrings of an input string. Our parallel algorithm in the multi-core shared memory setting has more than 600 % scaling performance on 16 threads. Our approach uses following five ideas which can be applied to other motif search problems as well:

**Efficient neighborhood generation:** We show that it is enough to consider the neighbors which are at a distance exactly *d* from all possible substrings of the input strings. This works because the neighbors at a lesser distance are also included in the neighborhood of some other substrings.

**Compact representation using wildcard characters:** We represent all possible neighbors which are due to an insertion or a substitution at a position by a single neighbor using a wildcard character at the same position. This compact representation of the candidate motifs in the neighborhood requires less space.

**Avoiding duplication of candidate motifs:** Our algorithm uses several rules to avoid duplication in candidate motifs and we prove that our technique generates neighborhood that is nearly duplication free. In other words, our neighborhood generation technique does not spend a lot of time generating neighbors that have already been generated.

**Trie based data structure for storing compact motifs:** We use a trie based data structure to efficiently store the neighborhood. This not only simplifies the removal of duplicate neighbors but also helps in outputting the final motifs in sorted order using a depth first search traversal of the trie.

**Modified radix-sort for compact motifs:** Our parallel algorithm stores the compact motifs in an array and uses a modified radix-sort algorithm to sort them. Use of arrays instead of tries simplifies updating the set of candidate motifs by multiple threads.

## Methods

In this section we introduce some notations and observations.

An (*l,d*)-friend of a *k*-mer *L* is an *l*-mer at an exact distance of *d* from *L*. Let *F*_*l,d*_(*L*) denote the set of all (*l,d*)-friends of *L*. An (*l,d*)*-neighbor* of a *k*-mer *L* is an *l*-mer at a distance of atmost *d* from *L*. Let *N*_*l,d*_(*L*) denote the set of all (*l,d*)-neighbors of *L*. Then

1 $$\begin{array}{*{20}l} N_{l,d}(L) = \cup_{t = 0}^{d} F_{l,t}(L).  \end{array} $$

For a string *S* of length *m*, an (*l,d*)*-motif* of *S* is an *l*-mer at a distance atmost *d* from some substring of *S*. Thus an (*l,d*)-motif of *S* is an (*l,d*)-neighbor of atleast one substring *S*_*j,k*_=*S*_*j*_*S*_*j*+1_…*S*_*j*+*k*−1_ where *k*∈[*l*−*d,l*+*d*]. Therefore, the set of (*l,d*)-motifs of *S*, denoted by *M*_*l,d*_(*S*), is given by

2 $$\begin{array}{*{20}l} M_{l,d}(S) = \cup_{k=l-d}^{l+d} \cup_{j=1}^{m-k+1} N_{l,d}(S_{j,k}).  \end{array} $$

For a collection of strings $\mathcal {S} = \{S^{(1)}, S^{(2)}, \ldots, S^{(m)}\}$, a (common) (*l,d*)-motif is an *l*-mer at a distance atmost *d* from atleast one substring of each *S*^(*i*)^. Thus the set of (common) (*l,d*)-motifs of $\mathcal {S}$, denoted by $M_{l,d}(\mathcal {S})$, is given by

3 $$\begin{array}{*{20}l} M_{l,d}(\mathcal{S}) = \cap_{i=1}^{n} M_{l,d}(S^{(i)}).  \end{array} $$

One simple way of computing *F*_*l,d*_(*L*) is to grow the friendhood of *L* by one distance at a time for *d* times and to select only the friends having length *l*. Let *G*(*L*) denote the set of strings obtained by one edit operation on *L* and $G(\{L_{1}, L_{2}, \ldots, L_{r}\}) = \cup _{t=1}^{r} G(L_{t})$. If *G*^1^(*L*)=*G*(*L*), and for *t*>1, *G*^*t*^(*L*)=*G*(*G*^*t*−1^(*L*)) then

4 $$\begin{array}{*{20}l} F_{l,d}(L) = \{x \in G^{d}(L) : |x| = l\}.  \end{array} $$

Using Eqs. (1), (2), (3) and (4), Pathak et al. [13] gave an algorithm that stores all possible candidate motifs in an array of size |*Σ*|^*l*^. However the algorithm is inefficient in generating the neighborhood as the same candidate motif is generated by several combinations of the basic edit operations. Also, the *O*(|*Σ*|^*l*^) memory requirement makes the algorithm inapplicable for larger instances. In this paper we mitigate these two limitations.

### Efficient neighborhood generation

We now give a more efficient algorithm to generate the (*l,d*)-neighborhood of all possible *k*-mers of a string. Instead of computing (*l,t*)-friendhood for all 0≤*t*≤*d*, we compute only the exact (*l,d*)-friendhood.

#### **Lemma1**.

$M_{l,d}(S) = \cup _{k=l-d}^{l+d} \cup _{j=1}^{m-k+1} F_{l,d}(S_{j,k})$.

#### *Proof*.

Consider the *k*-mer *L*=*S*_*j,k*_. If *k*=*l*+*d* then we need *d* deletions to make *L* an *l*-mer. There cannot be any (*l,t*)-neighbor of *L* for *t*<*d*. Thus

5 $$\begin{array}{*{20}l} \cup_{t = 0}^{d} F_{l,t}(S_{j,l+d}) = F_{l,d}(S_{j,l+d}).  \end{array} $$

Suppose *k*<*l*+*d*. Any (*l,d*−1)-neighbor *B* of *L* is also an (*l,d*)-neighbor of *L*^′^=*S*_*j,k*+1_ because *ED*(*B,L*^′^)≤*ED*(*B,L*)+*ED*(*L,L*^′^)≤(*d*−1)+1=*d*. Thus

$$\begin{array}{*{20}l} \cup_{t=0}^{d} F_{l,t}(S_{j,k}) \subseteq F_{l,d}(S_{j,k}) \bigcup \cup_{t=0}^{d} F_{l,t}(S_{j,k+1}) \end{array} $$

which implies that

6 $$\begin{array}{*{20}l} \cup_{r=k}^{k{+}1}\cup_{t{=}0}^{d} F_{l,t}(S_{j,r}) = F_{l,d}(S_{j,k}) \bigcup \cup_{t=0}^{d} F_{l,t}(S_{j,k{+}1}).  \end{array} $$

Applying (6) repeatedly for *k*=*l*−*d,l*−*d*+1,…,*l*+*d*−1, along with (5) in (1) and (2) gives the result of the lemma.

We generate *F*_*l,d*_(*S*_*j,k*_) in three phases: we apply *δ* deletions in the first phase, *β* substitutions in the second phase, and *α* insertions in the final phase, where *d*=*δ*+*α*+*β* and *l*=*k*−*δ*+*α*. Solving for *α,β,δ* gives max{0,*q*}≤*δ*≤(*d*+*q*)/2, *α*=*δ*−*q* and *β*=*d*−2*δ*+*q* where *q*=*k*−*l*. In each of the phases, the neighborhood is grown by one edit operation at a time.

### Compact motifs

The candidate motifs in *F*_*l,d*_(*S*_*j,k*_) are generated in a compact way. Instead of inserting each character in *Σ* separately at a required position in *S*_*j,k*_, we insert a new character ∗∉*Σ* at that position. Similarly, instead of substituting a character *σ*∈*S*_*j,k*_ by each *σ*^′^∈*Σ*∖{*σ*} separately, we substitute *σ* by ∗. The motifs common to all strings in $\mathcal {S}$ is determined by using the usual definition of union and the following definition of intersection on compact strings *A,B*∈(*Σ*∪{∗})^*l*^ in (3):

7 $$\begin{array}{@{}rcl@{}} \arraycolsep=1pt A {\cap} B = \left\{ \begin{array}{ll} \emptyset & \text{if}~ \exists j~\text{s.t.}~A_{j}, B_{j} \in \Sigma, A_{j} \ne B_{j}\\ \sigma_{1}\sigma_{2}\ldots \sigma_{l} & \text{else, where}~\sigma_{j} = \left\{ \begin{array}{ll} b_{j} & \text{if}~a_{j} {=} * \\ a_{j} & \text{if}~b_{j} {=} *. \end{array} \right. \end{array} \right.  \end{array} $$

### Trie for storing compact motifs

We store the compact motifs in a trie based data structure which we call a *motif trie*. This helps implement the intersection defined in (7). Each node in the motif trie has atmost |*Σ*| children. The edges from a node *u* to its children *v* are labeled with mutually exclusive subsets *label*(*u,v*)⊆*Σ*. An empty set of compact motifs is represented by a single root node. A non-empty trie has *l*+1 levels of nodes, the root being at level 0. The trie stores the *l*-mer *σ*_1_*σ*_2_…*σ*_*l*_, all *σ*_*j*_∈*Σ*, if there is a path from the root to a leaf where *σ*_*j*_ appears in the label of the edge from level *j*−1 to level *j*.

**Fig. 1:**
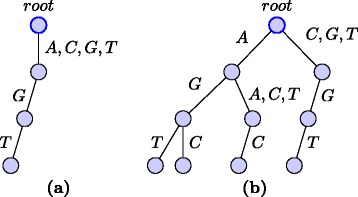
Inserting into motif trie for *Σ*={*A,C,G,T*} and *l*=3. **a** After inserting ∗*G* *T* into empty trie. **b** After inserting another string *A*∗*C*

For each string $S=\mathcal {S}^{(i)}$ we keep a separate motif trie *M*^(*i*)^. Each compact neighbor *A*∈*F*_*l,d*_(*S*_*j,k*_) is inserted into the motif trie recursively as follows. We start with the root node where we insert *A*_1_*A*_2_…*A*_*l*_. At a node *u* at level *j* where the prefix *A*_1_*A*_2_…*A*_*j*−1_ is already inserted, we insert the suffix *A*_*j*_*A*_*j*+1_…*A*_*l*_ as follows. If *A*_*j*_∈*Σ* we insert *A*^′^=*A*_*j*+1_*A*_*j*+2_…*A*_*l*_ to the children *v* of *u* such that *A*_*j*_∈*label*(*u,v*). If *label*(*u,v*)≠{*A*_*j*_}, before inserting we make a copy of subtrie rooted at *v*. Let *v*^′^ be the root of the new copy. We make *v*^′^ a new child of *u*, set *label*(*u,v*^′^)={*A*_*j*_}, remove *A*_*j*_ from *label*(*u,v*), and insert *A*^′^ to *v*^′^. On the other hand if *A*_*j*_=∗ we insert *A*^′^ to each children of *u*. Let *T*=*Σ* if *A*_*j*_=∗ and *T*={*A*_*j*_} otherwise. Let *R*=*T*∖∪_*v*_*label*(*u,v*). If *T*≠*∅* we create a new child *v*^′^ of *u*, set *label*(*u,v*^′^)=*R* and recursively insert *A*^′^ to *v*^′^. Figure 1 shows examples of inserting into the motif trie.

**Fig. 2:**
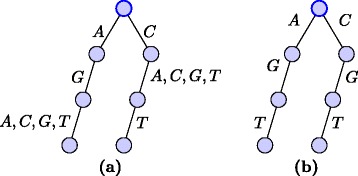
Intersection of motif tries. **a** Trie for *A* *G*∗∪*C*∗*T*. **b** Intersection of trie in Fig. 1 b and trie in Fig. 2 **a**



We also maintain a motif trie $\mathcal {M}$ for the common compact motifs found so far, starting with $\mathcal {M} = M^{(1)}$. After processing string *S*^(*i*)^ we intersect the root of *M*^(*i*)^ with the root of $\mathcal {M}$. In general a node *u*_2_∈*M*^(*i*)^ at level *j* is intersected with a node $u_{1} \in \mathcal {M}$ at level *j* using the procedure shown in Algorithm 1. Figure 2 shows an example of the intersection of two motif tries.

The final set of common motifs is obtained by a depth-first traversal of $\mathcal {M}$ outputting the label of the path from the root whenever a leaf is traversed. An edge (*u,v*) is traversed separately for each *σ*∈*label*(*u,v*).

### Efficient compact neighborhood generation

A significant part of the time taken by our algorithm is in inserting compact neighbors into the motif trie as it is executed for each neighbor in the friendhood. Our efficient neighborhood generation technique and the use of compact neighbors reduce duplication in neighborhood but do not guarantee completely duplication free neighborhood. In this section, we design few simple rules to reduce duplication further. Later we will see that these rules are quite close to the ideal as we will prove that the compact motif generated after skipping using the rules, are distinct if all the characters in the input string are distinct.

To differentiate multiple copies of the same compact neighbor, we augment it with the information about how it is generated. This information is required only in the proof and is not used in the actual algorithm. Formally, each compact neighbor *L* of a *k*-mer *S*_*j,k*_ is represented as an ordered tuple 〈*S*_*j,k*_,*T*〉 where *T* denotes the sequence of edit operations applied to *S*_*j,k*_. Each edit operation in *T* is represented as a tuple 〈*p,o*〉 where *p* denotes the position (as in *S*) where the edit operation is applied and *o*∈{*D,R,I*} denotes the type of the operation – deletion, substitution and insertion, respectively. At each position there can be one deletion or one substitution but one or more insertions. The tuples in *T* are sorted lexicographically with the natural order for *p* and for *o*, *D*<*R*<*I*.

**Fig. 3:**
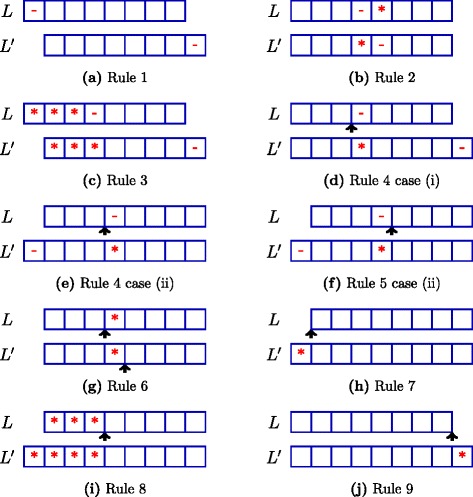
Construction of *L* ^′^ under different rules in the proof of Lemma 2. Insertions are shown using arrows, deletions using − and substitutions using ∗. Rule 5 case (i) is similar to Rule 4 case (i)

The rules for skipping compact neighbors are given in Table 2. Rule 1 applies when *S*_*j,k*_ is not the rightmost *k*-mer and the current edit operation deletes the leftmost base of *S*_*j,k*_, *i.e.*, *S*_*j*_. Rule 2 applies when the current edit operation substitutes a base just after a base that was already deleted. Rule 3 skips the neighbor which is generated from a *k*-mer except the rightmost by deleting a base and substituting all bases before it. Rules 4–9 apply when the current operation is an insertion. Rule 4,6 apply when the insertion is just before a deletion and a substitution, respectively. Rule 5 applies when the insertion is just after a deletion. Rule 7,8 apply when the *k*-mer is not the leftmost. Rule 7 applies when the insertion is at the leftmost position and Rule 8 applies when all bases before the position of insertion are already substituted. Rule 9 applies when the *k*-mer is not the rightmost and the insertion is at the right end. The first in each pair of the figures in Fig. 3 illustrates the situation where the corresponding rule applies.

**Table 2:** Conditions for skipping motif *L*=〈*M,S*_*j,k*_,*T*〉

Rule	Conditions (in all rules *t*≥0)
1	(*j*+*k*≤*m*)∧〈*j,D*〉∈*T*
2	{〈*j*+*t,D*〉,〈*j*+*t*+1,*R*〉}⊆*T*
3	(*j*+*k*≤*m*)∧{〈*j,R*〉,〈*j*+1,*R*〉,…,〈*j*+*t,R*〉,〈*j*+*t*+1,*D*〉}⊆*T*
4	{〈*j*+*t,D*〉,〈*j*+*t,I*〉}⊆*T*
5	{〈*j*+*t,D*〉,〈*j*+*t*+1,*I*〉}⊆*T*
6	{〈*j*+*t,R*〉,〈*j*+*t,I*〉}⊆*T*
7	(*j*>1)∧〈*j,I*〉∈*T*
8	(*j*>1)∧{〈*j,R*〉,〈*j*+1,*R*〉,…,〈*j*+*t,R*〉,〈*j*+*t*+1,*I*〉}⊆*T*
9	(*j*+*k*≤*m*)∧〈*j*+*k,I*〉∈*T*

Let $\bar {M}_{l,\,d}(S)$ denote the multi-set of tuples for the compact motifs of *S* that were not skipped by our algorithm using the rules in Table 2 and *M*_*l*, *d*_(*S*) be the set of compact motifs generated by (3). Let *Γ*(〈*S*_*j*, *k*_,*T*〉) be the resulting string when the operations in *T* are applied to *S*_*j*, *k*_ and *Γ*(*Z*)=∪_*L*∈*Z*_*Γ*(*L*).

#### **Lemma2**.

$\Gamma (\bar {M}_{l,d}(S)) = M_{l,d}(S)$.

#### *Proof*.

By construction, $\Gamma (\bar {M}_{l,d}(S)) \subseteq M_{l,d}(S)$. We show $M_{l,d}(S) \subseteq \Gamma (\bar {M}_{l,d}(S))$ by giving a contradiction when $M_{l,d}(S) \setminus \Gamma (\bar {M}_{l,d}(S)) \ne \emptyset $.

We define an order on the compact neighbors $\phantom {\dot {i}\!}L_{1} = \langle {S_{j_{1},k_{1}}, T_{1}}\rangle $ and $\phantom {\dot {i}\!}L_{2} = \langle {S_{j_{2},k_{2}}, T_{2}}\rangle $ as follows: *L*_1_<*L*_2_ if *Γ*(*L*_1_)<*Γ*(*L*_2_) and *L*_2_<*L*_1_ if *Γ*(*L*_2_)<*Γ*(*L*_1_). When *Γ*(*L*_1_)=*Γ*(*L*_2_) we have *L*_1_<*L*_2_ if and only if (*k*_1_<*k*_2_)∨((*k*_1_=*k*_2_)∧(*p*_1_<*p*_2_))∨((*k*_1_=*k*_2_)∧(*p*_1_=*p*_2_)∧(*o*_1_<*o*_2_)) where 〈*p*_1_,*o*_1_〉∈*T*_1_,〈*p*_2_,*o*_2_〉∈*T*_2_ are the leftmost edit operations where *T*_1_,*T*_2_ differ.

Suppose $M \in M_{l,d}(S) \setminus \Gamma (\bar {M}_{l,d}(S))$. Let *L*=〈*S*_*j*, *k*_,*T*〉 be the largest (in the order defined above) tuple skipped by our algorithm such that *Γ*(*L*)=*M*. For each *r*=1,…,9 we show a contradiction that if *L* is skipped by Rule *r* then there is another $\phantom {\dot {i}\!}L'=\langle {S_{j',\,k'},T'}\rangle $ with the same number of edit operations and *Γ*(*L*^′^)=*M* but *L*<*L*^′^. Figure 3 illustrates the choice of *L*^′^ under different rules.

Rule 1. Here *j*+*k*≤*m* and 〈*j,D*〉∈*T*. Consider *T*^′^=(*T*∖{〈*j,D*〉})∪{*j*+*k,D*}, and *j*^′^=*j*+1,*k*^′^=*k*.

Rule 2. Consider *T*^′^=*T*∖{〈*j*+*t,D*〉,〈*j*+*t*+1,*R*〉}∪{〈*j*+*t,R*〉,〈*j*+*t*+1,*D*〉}, and *j*^′^=*j,k*^′^=*k*.

Rule 3. *T*^′^=*T*∖{〈*j,R*〉,〈*j*+*t*+1,*D*〉}∪{〈*j*+*t*+1,*R*〉,〈*j*+*k,D*〉}, *j*^′^=*j*+1,*k*^′^=*k*.

Rule 4. For this and subsequent rules *k*<*l*+*d* as there is atleast one insertion and hence *k*^′^ could possibly be equal to *k*+1. We consider two cases. Case (i) *j*+*k*≤*m*: *T*^′^=*T*∖{〈*j*+*t,D*〉,〈*j*+*t,I*〉}∪{〈*j*+*t,R*〉,〈*j*+*k,D*〉}, *j*^′^=*j,k*^′^=*k*+1. Case (ii) *j*+*k*=*m*+1: Here deletion of *S*_*j*_ is allowed by Rule 1. *T*^′^=*T*∖{〈*j*+*t,D*〉,〈*j*+*t,I*〉}∪{〈*j*−1,*D*〉,〈*j*+*t,R*〉}, *j*^′^=*j*−1,*k*^′^=*k*+1.

Rule 5. The same argument for Rule 4 applies considering 〈*j*+*t*+1,*I*〉 instead of 〈*j*+*t,I*〉.

Rule 6. *T*^′^=*T*∖{〈*j*+*t,I*〉}∪{〈*j*+*t*+1,*I*〉}, and *j*^′^=*j,k*^′^=*k*.

Rule 7. *T*^′^=*T*∖{〈*j,I*〉}∪{〈*j*−1,*R*〉}, *j*^′^=*j*−1,*k*^′^=*k*+1.

Rule 8. *T*^′^=*T*∖{〈*j*+*t,I*〉}∪{〈*j*−1,*R*〉}, *j*^′^=*j*−1,*k*^′^=*k*+1.

Rule 9. *T*^′^=*T*∖{〈*j*+*k,I*〉}∪{〈*j*+*k,R*〉}, *j*^′^=*j,k*^′^=*k*+1.

Consider two compact motifs $\phantom {\dot {i}\!}M_{1} = \langle {S_{j_{1},k_{1}}, T_{1}}\rangle $ and $\phantom {\dot {i}\!}M_{2} = \langle {S_{j_{2},k_{2}}, T_{2}}\rangle $ in $\bar {M}_{l,d}(S)$. For *q*∈{1,2}, let $\left \langle {p_{q}^{(1)}, o_{q}^{(1)}}\right \rangle, \left \langle {p_{q}^{(2)}, o_{q}^{(2)}}\right \rangle, \ldots, \left \langle {p_{q}^{(d)}, o_{q}^{(d)}}\right \rangle $ be the sequence of edit operations in *T*_*q*_ arranged in the order as the neighbors are generated by our algorithm, and the intermediate neighbors be $L_{q}^{(h)} = \left \langle S_{j_{q},k_{q}}, \left \{\left \langle {p_{q}^{(1)}, o_{q}^{(1)}}\right \rangle,\right.\right.\left.\left. \left \langle {p_{q}^{(2)}, o_{q}^{(2)}}\right \rangle, \ldots, \left \langle {p_{q}^{(h)}, o_{q}^{(h)}}\right \rangle \right \} \right \rangle $ for all *h*=1,2,…,*d*. We also denote the initial *k*-mer as a neighbor $L_{q}^{(0)} = \langle {S_{j_{q},k_{q}}, \emptyset }\rangle $.

#### **Lemma3**.

If *S*_*j*_s are all distinct and $\Gamma \left (L_{1}^{(h)}\right) = \Gamma \left (L_{2}^{(h)}\right)$ for 1≤*h*≤*d* then $\left \langle {p_{1}^{(h)}, o_{1}^{(h)}}\right \rangle = \left \langle {p_{2}^{(h)}, o_{2}^{(h)}}\right \rangle $ and $\Gamma \left (L_{1}^{(h-1)}\right) = \Gamma \left (L_{2}^{(h-1)}\right)$.

#### *Proof*.

To simplify the proof, we use *p*_*q*_,*o*_*q*_,*L*_*q*_ to denote $p_{q}^{(h)}, o_{q}^{(h)}, L_{q}^{(h)}$, respectively, for all *q*∈{1,2}. Without loss of generality we assume *p*_1_≤*p*_2_.

As *p*_1_,*p*_2_ are positions in *S*, it would be enough to prove 〈*p*_1_,*o*_1_〉=〈*p*_2_,*o*_2_〉 because that would imply $\Gamma \left (L_{1}^{(h-1)}\right) = \Gamma \left (L_{2}^{(h-1)}\right)$.

If 〈*p*_1_,*o*_1_〉≠〈*p*_2_,*o*_2_〉 then either (a) *o*_1_=*o*_2_ and *p*_1_<*p*_2_ or (b) *o*_1_≠*o*_2_ and *p*_1_≤*p*_2_, giving us the following 9 possible cases. We complete the proof by giving a contradiction in each of these 9 cases:

**Table:** 

**Case**	***o***_**1**_	***o***_**2**_	**cond.**	**Case**	***o***_**1**_	***o***_**2**_	**cond.**	**Case**	***o***_**1**_	***o***_**2**_	**cond.**
1	*D*	*D*	*p*_1_<*p*_2_	4	*R*	*D*	*p*_1_≤*p*_2_	7	*I*	*D*	*p*_1_≤*p*_2_
2	*D*	*R*	*p*_1_≤*p*_2_	5	*R*	*R*	*p*_1_<*p*_2_	8	*I*	*R*	*p*_1_≤*p*_2_
3	*D*	*I*	*p*_1_≤*p*_2_	6	*R*	*I*	*p*_1_≤*p*_2_	9	*I*	*I*	*p*_1_<*p*_2_

***Cases 2, 3, 4, 7***

Our algorithm applies edit operations in phases: first deletions, followed by substitutions and finally insertions. In all these cases, one of *Γ*(*L*_1_),*Γ*(*L*_2_) does not have any ∗ because only deletions have been applied so far and the other has at least one ∗ because a substitution or an insertion has been applied. This implies *Γ*(*L*_1_)≠*Γ*(*L*_2_), a contradiction.

***Case 1***

*L*_2_ has $S_{p_{2}}$ deleted. As *Γ*(*L*_1_)=*Γ*(*L*_2_), $S_{p_{2}}$ must have been deleted in some operation prior to reaching *L*_1_. As the deletions are applied in order, left to right, we must have *p*_1_=*p*_2_ which is a contradiction.

***Case 5***

**Fig. 4:**
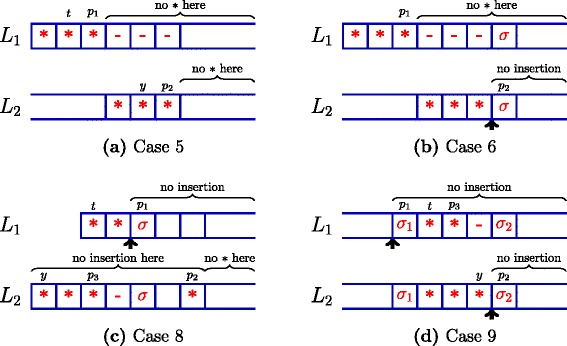
Proof of uniqueness (Lemma 2). Subfigures a,b,c,d illustrates the cases 5,6,7,8,9 respectively

This case has been illustrated in Fig. 4a. *L*_1_ has no substitution at a position >*p*_1_ and no insertion at all. The ∗ at *p*_2_ in *L*_2_ must be matched with the ∗ at *p*_1_ in *L*_1_ and as the characters in *S* are distinct, all of $S_{p_{1}+1},\ldots,S_{p_{2}}$ cannot appear in *L*_1_ and hence must be deleted in *L*_1_.

Now for each *t*<*p*_1_, right to left, and *y*=*t*+*p*_2_−*p*_1_, we have the following: *S*_*y*_ is either deleted or substituted in *L*_1_, which implies that *S*_*y*_ must be substituted in *L*_2_ as the deletion of *S*_*y*_ in *L*_2_ is prohibited by Rule 2, and finally to match this ∗ in *L*_2_, *S*_*t*_ must be substituted in *L*_1_ as *S*_*t*_ cannot be deleted in *L*_1_, again by Rule 2.

But this makes Rule 3 applicable to *L*_1_ and *L*_1_ must have been skipped. This is a contradiction.

***Case 6***

By Rule 9 the insertion in *L*_2_ cannot be at the rightmost position and hence *L*_2_ must have at least one character after the insertion. By Rules 4 and 6, $S_{p_{2}}$ must not be deleted or substituted in *L*_2_ and hence it must not be deleted or substituted in *L*_1_ either. Thus *p*_1_<*p*_2_. There cannot be any insertion or substitution at a position >*p*_1_ in *L*_1_. Thus the ∗ due to the insertion at *p*_2_ in *L*_2_ must be matched by the ∗ due to the substitution at *p*_1_ in *L*_1_ and all of $S_{p_{1}+1},\ldots,S_{p_{2}-1}$ must be deleted in *L*_1_.

By Rule 7, $S_{p_{2}}$ cannot be the leftmost in $S_{j_{2},k_{2}}$. So we consider $S_{p_{2}-1}$ in *L*_1_,*L*_2_. It is either deleted or substituted in *L*_1_ and hence by Rule 5, it must be substituted in $S_{p_{2}}$ (there can be multiple insertions at *p*_2_ in *L*_2_ but that does not affect this argument). To match this ∗, $S_{p_{1}-1}$ must be substituted in *L*_1_.

Using a similar argument as in Case 5, *S*_*t*_ must be substituted in *L*_1_ for each *t*<*p*_1_−1. But this again makes Rule 3 applicable to *L*_1_ and *L*_1_ must have been skipped, which is not possible. This case has been illustrated in Fig. 4b.

***Case 8***

Due to Rules 4, 6 and 9, $S_{p_{1}}$ must not be deleted or substituted in *L*_1_ and hence it must not be deleted or substituted in *L*_2_ either. Thus *p*_1_<*p*_2_. The ∗ due to the insertion in *L*_1_ must be matched by a substitution at *p*_3_<*p*_1_ such that all of $S_{p_{3}+1}, \dots, S_{p_{1}-1}$ are deleted in *L*_2_.

By Rule 7, *p*_1_ cannot be the leftmost in *L*_1_. For each *t*<*p*_1_, right to left, and *y*=*t*+*p*_3_−*p*_1_, we have the following: *S*_*y*_ is substituted in *L*_1_ because as the deletion of *S*_*y*_ in *L*_1_ is prohibited by Rules 2 and 5, *S*_*y*_ must be substituted in *L*_2_ again by Rule 2, and to match this ∗, *S*_*t*_ must be substituted in *L*_1_.

But this makes Rule 8 applicable to *L*_1_ and *L*_1_ must have been skipped which is not possible. This case has been illustrated in Fig. 4c.

***Case 9***

This case has been illustrated in Fig. 4d. Due to Rules 4, 6 and 9, $S_{p_{1}},S_{p_{2}}$ must not be deleted or substituted in *L*_1_,*L*_2_. The insertion at *p*_2_ in *L*_2_ must be matched by a substitution at a position *p*_3_ in *L*_1_ such that *p*_1_<*p*_3_<*p*_2_ and all of $S_{p_{3}+1},\ldots,S_{p_{2}-1}$ must be deleted in *L*_1_.

Now for each position *y*, from right to left, where *p*_1_<*y*<*p*_2_, *S*_*y*_ is either deleted or substituted in *S*_1_, *S*_*y*_ cannot be deleted in *L*_2_ by Rules 2 and 5 and hence must be substituted in *L*_2_, which again must be matched by a substitution at a position *t* in *L*_1_ such that *p*_1_<*t*<*p*_3_. However this is impossible as the number of possible *y*s is larger than the number of possible *t*s.

If all *S*_*j*_s are distinct and *Γ*(*M*_1_)=*Γ*(*M*_2_) then applying Lemma 3 repeatedly for *h*=*d,d*−1,…,0 gives us the fact that starting *k*-mers $S_{j_{1},k_{1}},S_{j_{2},k_{2}}$ as well as the corresponding edit operations in *T*_1_,*T*_2_ for *M*_1_,*M*_2_ must be the same. This is another way of stating the following theorem.

#### **Theorem1**.

If *S*_*j*_s are all distinct then $\bar {M}_{l,d}(S)$ is duplication free.

**Fig. 5:**
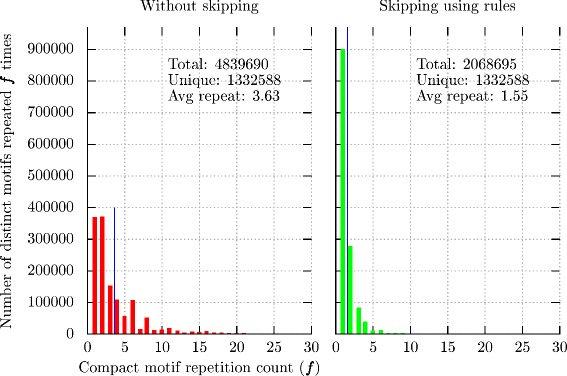
Histogram of number of times a motif is repeated with and without using the skipping rules 1–9

In general *S*_*j*_s are not distinct. However, as the input strings are random, the duplication due to repeated characters are limited. On instance (11,3) our algorithm generates each compact motif, on an average, 1.55 times using the rules compared to 3.63 times without the rules (see Fig. 5).

**Implementation** To track the deleted characters, instead of actually deleting we substitute them by a new symbol − not in *Σ*^′^. We populate the motif trie *M*^(*i*)^ by calling *genAll*(*S*^(*i*)^) given in Algorithm 2. Rules 1–8 are incorporated in *G*(*L,j*,*δ*,*β*,*α*), *H*(*L,j*,*β*,*α*) and *I*(*L,j*,*α*) which are shown in Algorithms 3, 4, and 5, respectively where *sub*(*L,j*,*σ*) substitutes *L*_*j*_ by *σ* and *ins*(*L,j*,*σ*) inserts *σ* just before *L*_*j*_.









### Modified radix-sort for compact motifs

A simpler data structure alternative to tries for storing compact motifs could be an array. However, it becomes difficult to compute the intersection in (3) as defined in (7) when the compact motifs are stored in arrays. One straight-forward solution is to first expand the ∗s in the compact motifs, then sort the expanded motifs and finally compute the intersection by scanning through the two sorted arrays. This, to a great extent, wipes out the advantage using the ∗s in the compact motifs. However, we salvage execution time by executing a modified radix-sort that simultaneously expands and sorts the array of compact motifs: Compact-Radix-Sort(*A,l*) where the first parameter *A* represents the array of compact motifs and the second parameter represents the number of digits of the elements in *A* which is equal to the number of base positions *l* in a motif.

As in the standard radix-sort, our algorithm uses *l* phases, one for each base position in the motif. In the *i*th phase it sorts the motifs using bucket sort on the *i*th base of the motifs. However, in case of compact motifs, for each ∗ at a base position, the bucket counters for all *σ*∈*Σ* are incremented. While reordering the motifs as per the bucket counts, if there is a ∗ at *i*th base position of a motif, |*Σ*| copies of the motif are created and they are placed at appropriate locations in the array after finalizing the correct *σ* for the ∗. The details are given in Algorithm 6. In each phase a bucket counter *B* and a cumulative counter *C* are used. The temporary array *T* stores the partially expanded motifs from the current phase.

**Discussion** We did an experiment to compare the time taken by the two approaches – (i) using the expanded motifs, *i.e.*, without using the wildcard character, and (ii) using compact motifs and sorting them using Compact-Radix-Sort. For a single input string of instance (16,3), the first approach generated in 24.4 s 198,991,822 expanded motifs in which 53,965,581 are unique. The second approach generated in 13.7 s 11,474,938 compact motifs with the same number of unique expanded motifs. This shows the effectiveness of the second approach.



### Parallel algorithm

We now give our parallel algorithm in the multi-core shared memory setting. To process each input sequence *S*^(*i*)^ the algorithm uses *p*+1 threads. The main thread first prepares the workload for other *p* threads. A workload involves the generation of the neighborhood for a *k*-mer of *S*^(*i*)^, where *l*−*d*≤*k*≤*l*+*d*. There are total $\sum _{k=l-d}^{l+d} (m-k+1) = (2d+1)(m-l+1)$ workloads. The number of neighbors generated in the workloads are not the same due to the skipping of some neighbors using rules 1–9. For load balancing, we randomly and evenly distribute workloads to threads. Each thread first generates all the compact motifs in its workloads and then sort them using Compact-Radix-Sort. If *i*>2 then it removes all neighbors not present in *M*^(*i*−1)^ which is the set of common motifs of *S*^(1)^,*S*^(2)^,…,*S*^(*i*−1)^. The master thread then merges the residue candidate motifs from all the *p* threads to compute *M*^(*i*)^. The merging takes place in log2*p* phases in a binary tree fashion where the *j*th phase uses $2^{\log _{2}{p} - j}$ threads each merging two sorted arrays of motifs.

## Results and discussion

We implemented our algorithms in C++ and evaluated on a Dell Precisions Workstation T7910 running RHEL 7.0 on two sockets each containing 8 Dual Intel Xeon Processors E5-2667 (8C HT, 20 MB Cache, 3.2 GHz) and 256 GB RAM. For our experiments we used only one of the two sockets. We generated random (*l,d*) instances according to Pevzner and Sze [2] and as described in the background section. For every (*l,d*) combination we report the average time taken by 4 runs. We compare the following four implementations:

- **EMS1:** A modified implementation of the algorithm in [13] which considered the neighborhood of only *l*-mers whereas the modified version considers the neighborhood of all *k*-mers where *l*−*d*≤*k*≤*l*+*d*.
- **EMS2:** A faster implementation of our sequential algorithm which uses tries for storing candidate motifs where each node of the trie stores an array of pointers to each children of the node. However, this makes the space required to store a tree node dependent on the size of the alphabet *Σ*.
- **EMS2M:** A slightly slower but memory efficient implementation of our sequential algorithm where each node of the trie keeps two pointers: one to the leftmost child and the other to the immediate right sibling. Access to the other children are simulated using the sibling pointers.
- **EMS2P:** Our parallel algorithm which uses arrays for storing motifs. We experimented with *p*=1,2,4,8,16 threads.

We run the four algorithms on the challenging instances (8,1), (12,2), (16,3) and on the instances (9,2), (11,3), (13,4) which are challenging for PMS and have been used for experimentation in [13]. We report the runtime and the memory usage of the four algorithms in Table 3.

**Table 3:** Comparison between EMS1 and three implementations of EMS2

Instance	Metric	EMS1	EMS2	EMS2M	EMS2P threads				
					1	2	4	8	16
(8,1)	time	0.11 s	0.13 s	0.12 s	0.09 s	0.08 s	0.06 s	0.05 s	0.06 s
	memory	2.69 MB	4.25 MB	3.17 MB	2.67 MB	3.20 MB	3.55 MB	6.02 MB	7.99 MB
(12,2)	time	19.87 s	15.60 s	16.62 s	2.71 s	1.94 s	1.44 s	0.89 s	0.55 s
	memory	34.28 MB	210.47 MB	126.91 MB	84.98 MB	104.60 MB	125.18 MB	142.82 MB	150.23 MB
(16,3)	time	1.74 h	23.73 m	26.79 m	3.73 m	2.32 m	1.38 m	48.58 s	36.93 s
	memory	8.39 GB	11.62 GB	6.97 GB	8.55 GB	10.21 GB	10.53 GB	9.84 GB	9.91 GB
(9,2)	time	10.84 s	1.72 s	3.02 s	1.12 s	0.96 s	0.78 s	0.49 s	0.35 s
	memory	3.44 MB	26.67 MB	17.04 MB	42.86 MB	57.76 MB	54.77 MB	59.85 MB	66.53 MB
(11,3)	time	33.48 m	1.91 m	3.57 m	45.85 s	30.78 s	19.68 s	13.49 s	9.78 s
	memory	92.86 MB	477.12 MB	313.33 MB	2.27 GB	2.63 GB	2.65 GB	2.55 GB	2.60 GB
(13,4)	time	-	1.08 h	1.76 h	44.03 m	26.16 m	14.51 m	8.62 m	6.82 m
	memory	-	8.26 GB	5.58 GB	149.60 GB	179.66 GB	180.13 GB	168.80 GB	172.74 GB

Time is in seconds (s), minutes (m) or hours (h). An empty cell implies the algorithm did not complete in the stipulated 72 h

Our efficient neighborhood generation enables our algorithm to solve instance (13,4) in less than two hours which EMS1 could not solve even in 3 days. The factor by which EMS2 takes more memory compared to EMS1 gradually decreases as instances become harder. As EMS2 stores 4 child pointers for *A,C,G,T* in each node of the motif trie whereas EMS2M simulates access to children using only 2 pointers, EMS2 is faster. Memory reduction in EMS2M is not exactly by a factor 2(=4/2) because we also keep a bit vector in each node to represent the subset of {*A,C,G,T*} a child corresponds to. The memory reduction would be significant for protein strings.

**Fig. 6:**
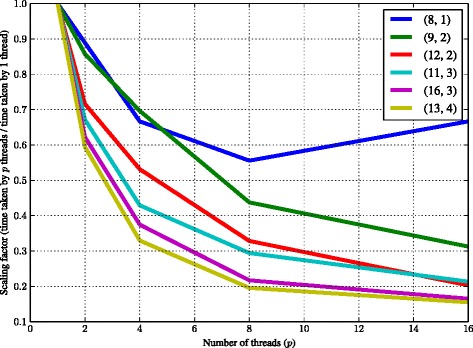
Scaling performance of our parallel algorithm

Our parallel algorithm EMS2P using one thread is significantly faster than the sequential algorithms EMS2 and EMS2M but uses more memory. This space-time trade off is due to the fact that the arrays are faster to access but the tries use lesser memory. Moreover, the repeated motifs are uniquely stored in a single leaf node in the trie but stored separately in the array. The scaling performance using multiple threads are shown in Fig. 6 where we plot the ratio of time taken by *p* threads to the time taken by a single thread on the Y-axis. The time required for handling 16 threads turns out to be costlier than actually processing the motifs in the smallest instance (8,1). We observe speed up consistent across other bigger instances. For example, instance (16,3) takes about 224 s using 1 thread and 37 s using 16 threads. This gives more than 600 % scaling performance using 16 threads.

## Conclusions

We presented several efficient sequential and parallel algorithms for the EMS problem. Our algorithms use some novel and elegant rules to explore the candidate motifs in such a way that only a small fraction of the candidate motifs are explored twice or more. In fact, we also proved that these rules are close to ideal in the sense that no candidate motif is explored twice if the characters in the input string are all distinct. This condition may not be practical and ideas from [14] can be used when the characters in the input string are repeated. Nevertheless, the rules help because the instances are randomly generated and the *k*-mers in the input string are not much frequent. The second reason for the efficiency of our sequential algorithms is the use of a trie based data structure to compactly store the motifs. Our parallel algorithm stores candidate motifs in an array and uses a modified radix-sort based method for filtering out invalid candidate motifs.

Our algorithms pushed up the state-of-the-art of EMS solvers to a state where the challenging instance (16,3) is solved in slightly more than half a minute using 16 threads. Future work could be to solve harder instances, including those involving protein strings, and possibly using many-core distributed algorithms.

## Additional file

Additional file 1 Expected number of spurious motifs. This file gives the expression for the expected number of spurious (*l,d*)-motifs in *n* random strings of length *m* from the alphabet *Σ*. (PDF 143 kb)
