# Supplementary material for: Efficient sequential and parallel algorithms for finding edit distance based motifs
Source: BMC Genomics. 2016 Aug 18;17(Suppl 4):465. doi: 10.1186/s12864-016-2789-9 (PMC5001234; doi:10.1186/s12864-016-2789-9)
Supplement: Additional file 1 — Expected number of spurious motifs. This file gives the expression for the expected number of spurious (l,d)-motifs in n random strings of length m from the alphabet Σ. (PDF 143 kb) [file 12864_2016_2789_MOESM1_ESM.pdf]

Expected Number of Spurious Motifs

We compute the expected number of spurious motifs, i.e., motifs occurring by chance, in n randomly generated strings each having m random characters from the alphabet Σ.

Let M be a motif of length l and L be an occurrence of M of length $l+q$ with δ deletions, β substitutions and α insertions where , $-d \le q \le d$. The number of such possible L is

$$N(\delta,\beta,\alpha) = \binom{l+q}{\delta}\binom{l+q-\delta}{\beta}\binom{l+q-\delta+\alpha}{\alpha}|\Sigma|^{\alpha}(|\Sigma|-1)^{\beta}$$

and the probability that a random L of length $l+q$ is a neighbor of M is

$$N(\delta,\beta,\alpha)/|\Sigma|^{l+q}.$$

As discussed in the section on methods in the main article, the possible number of deletions, substitutions and insertions (δ, β, α, respectively), for a d-neighbor are given by the following set of equations: $\max\{0,q\} \le \delta \le (d+q)/2, \alpha = \delta - q, \beta = d + q - 2\delta$. Thus, for any random L of length $l+q$, the probability that L is an occurrence of M is

$$P = \sum_{\delta=\max\{0,q\}}^{\frac{d+q}{2}} \frac{N(\delta, d+q-2\delta, \delta-q)}{|\Sigma|^{l+q}}.$$

There could be $(m-l-q+1)$ number of $(l+q)$-mers of a string S of length m. The probability that M does not occur in S is

$$R = \Pi_{q=-d}^{d}(1-P)^{m-l-q+1}.$$

The probability that M occurs in each of the input strings in $\mathcal{S} = \{S^{(1)}, S^{(2)}, \dots, S^{(n)}\}$ is $(1-R)^n$. Since M can be any arbitrary motif, the expected number of common (l,d)-motifs of $\mathcal{S}$ is

$$E(\mathcal{S}, l, d) = |\Sigma|^{l}(1-R)^{n}. \tag{1}$$

Table 1 shows the expected number of spurious motifs for $l \in [5, 21]$ and d upto $\max\{l-2, 13\}$, $n = 20$, $m = 600$ and $\Sigma = \{A, C, G, T\}$ computed using (1).

Table 1 Expected number of spurious motifs in random instances for n=20, m=600. Here, ∞ represents value $\geq$1.0e+7.

l	d=0	1	2	3	4	5	6	7	8	9	10	11	12	13
5	0.0	1024.0	1024.0	∞										
6	0.0	4096.0	4096.0	∞	∞									
7	0.0	14141.8	16384.0	∞	∞	∞								
8	0.0	**225.8**	65536.0	65536.0	∞	∞	∞							
9	0.0	0.0	262144.0	262144.0	∞	∞	∞	∞						
10	0.0	0.0	1047003.6	1048576.0	∞	∞	∞	∞	∞					
11	0.0	0.0	1332519.5	4194304.0	∞	∞	∞	∞	∞	∞				
12	0.0	0.0	**294.7**	1.678e+07	1.678e+07	∞	∞	∞	∞	∞	∞			
13	0.0	0.0	0.0	6.711e+07	6.711e+07	∞	∞	∞	∞	∞	∞	∞		
14	0.0	0.0	0.0	2.517e+08	2.684e+08	∞	∞	∞	∞	∞	∞	∞	∞	
15	0.0	0.0	0.0	2.749e+07	1.074e+09	∞	∞	∞	∞	∞	∞	∞	∞	∞
16	0.0	0.0	0.0	**139.1**	4.295e+09	4.295e+09	∞	∞	∞	∞	∞	∞	∞	∞
17	0.0	0.0	0.0	0.0	1.718e+10	1.718e+10	∞	∞	∞	∞	∞	∞	∞	∞
18	0.0	0.0	0.0	0.0	3.965e+10	6.872e+10	∞	∞	∞	∞	∞	∞	∞	∞
19	0.0	0.0	0.0	0.0	1.226e+08	2.749e+11	2.749e+11	∞	∞	∞	∞	∞	∞	∞
20	0.0	0.0	0.0	0.0	**35.8**	1.100e+12	1.100e+12	∞	∞	∞	∞	∞	∞	∞
21	0.0	0.0	0.0	0.0	0.0	4.333e+12	4.398e+12	∞	∞	∞	∞	∞	∞	∞
